# Spread of the cycles: a feedback perspective on the Anthropocene

**DOI:** 10.1098/rstb.2022.0254

**Published:** 2024-01-01

**Authors:** Timothy M. Lenton, Marten Scheffer

**Affiliations:** ^1^ Global Systems Institute, University of Exeter, Exeter EX4 4QE, UK; ^2^ Wageningen University, Wageningen NL-6700 AA, The Netherlands

**Keywords:** feedback, cycles, cultural evolution, revolution, Anthropocene, sustainability

## Abstract

What propelled the human ‘revolutions' that started the Anthropocene? and what could speed humanity out of trouble? Here, we focus on the role of reinforcing feedback cycles, often comprised of diverse, unrelated elements (e.g. fire, grass, humans), in propelling abrupt and/or irreversible, revolutionary changes. We suggest that differential ‘spread of the cycles' has been critical to the past human revolutions of fire use, agriculture, rise of complex states and industrialization. For each revolution, we review and map out proposed reinforcing feedback cycles, and describe how new systems built on previous ones, propelling us into the Anthropocene. We argue that to escape a bleak Anthropocene will require abruptly shifting from existing unsustainable ‘vicious cycles’, to alternative sustainable ‘virtuous cycles' that can outspread and outpersist them. This will need to be complemented by a revolutionary cultural shift from maximizing growth to maximizing persistence (sustainability). To achieve that we suggest that non-human elements need to be brought back into the feedback cycles underlying human cultures and associated measures of progress.

This article is part of the theme issue ‘Evolution and sustainability: gathering the strands for an Anthropocene synthesis’.

## 1. Introduction

How did humanity get into the Anthropocene? and how can humanity make it an Epoch to be proud of? These are huge questions with many disciplines and perspectives contributing to answering them. Here, we focus on the processes that determine which human systems—such as farming or capitalism—come to dominate and transform the world, and how one system can, sometimes abruptly and/or irreversibly, supersede another in a revolutionary change. An abrupt transformation to sustainability is now required worldwide to avoid the worst damages from climate change and nature loss, and to tackle rising global inequality. Hence, it is important to understand how current unsustainable human systems became dominant, as the same forces are probably keeping them resiliently in place. This can help point to the qualities that more sustainable systems need to possess to rapidly displace them.

There are at least three theoretical approaches to understanding which large-scale human systems come to dominate and transform the world. First is cultural evolution, which considers how information capable of affecting individuals' behaviour (i.e. culture) that is acquired from other humans, can change over time [1]. Second is complex adaptive systems theory, which emphasizes how flows of information through feedback loops can give rise to and affect the spread and persistence of different non-equilibrium sociocultural systems [2,3]. Third is the theory of long-run economic growth, which focuses on how changing feedbacks between human population, innovation and resources over time have produced transitions between different growth regimes [4–7]. Here, we seek to synthesize insights from these three approaches with a focus on identifying the reinforcing feedback loops behind past, and possible future, human revolutions.

Recently with colleagues, we introduced a ‘survival of the systems' framework [3] that attempted to bridge the evolution and complex systems approaches. This recognized that selection based solely on differential persistence [8] could be a plausible mechanism for the evolution of large-scale social [9,10] and ecological [11,12] systems. In essence, those systems with lower extinction rate or higher spread rate tend to come to dominate the world [9]. As a locus for selection, we focused on the irreducible self-amplifying or self-damping properties of feedback cycles, as these can be critical to determining the spread and persistence of the social-ecological systems containing them [3]. Innovation and selection at lower levels can provide a source of variation in feedback cycles at the (higher) system level [13]. Differential extinction tends to be a slow evolutionary mechanism, with a timescale of approximately 500 years which may apply to early societies, but is far too slow to explain e.g. the Industrial Revolution [10]. By contrast, some systems out-spreading other systems can be a faster evolutionary mechanism [14], intuitively closer to familiar measures of ‘fitness' based on growth and fecundity.

Here in ‘spread of the cycles', we make a further bridge to long-run economic growth theory, and we focus on identifying and visualizing key proposed self-amplifying feedback cycles which can affect the differential spread of major social-ecological systems—especially those that played a role in past ‘revolutions' that got us into the Anthropocene: the taming of fire, the Agricultural Revolution, the rise of complex states and the Industrial Revolution. This also draws on existing systems approaches to the evolution of societies, including: systems ecology's focus on auto-catalytic feedback cycles [15]; industrial ecology's identification of different socio-metabolic regimes [16]; archaeologist's identification of reinforcing feedbacks in the rise and fall of complex societies [17]; and comparative historian's inferences of causal relationships [18].

We recognize that there are a wide variety of hypotheses for each past revolution and often a shortage of data and models to engage in comparative hypothesis testing. Our aim is not to solve that problem, but rather to aid understanding by synthesizing and framing existing hypotheses and insights within a common framework of causal feedback loop diagrams. This is a widely recognized early step in systems thinking towards more formalized systems modelling and hypothesis testing [19], and we were struck by the lack of it in relevant literature—with notable exceptions [20]. For some specific transitions more advanced progress is being made, e.g. through the construction and calibration of models from cross-cultural data [21], or formalized hypothesis testing using large historical datasets [18].

For each past revolution, we briefly consider how to interpret key feedbacks from a cultural evolution perspective. Then we turn to focus on identifying alternative, more sustainable feedback cycles that could play an urgent role in getting us towards sustainability. Here, we draw on existing systems approaches to understanding sustainability transformation, including: leverage points [19]; limits to growth [22]; industrial ecology [23]; ecological economics [24]; and transitions research [25].

We start by orienting our approach with respect to existing theories and introducing some key concepts.

## 2. Theoretical foundations and relationship to existing work

We take a pluralist approach to understanding which human systems come to dominate and transform the world, drawing on several explanatory frameworks.

Cybernetics long-ago established that a single system with a source of variation within it can gain persistence-enhancing feedback properties, through a series of repeated trials over time [26–28] (without requiring a population of systems). Darwin introduced ‘population thinking': looking at a population of items of different types (subpopulations) with the frequency of types changing over time [29]. Within population thinking, there are nested explanatory frameworks [30]: a population is *evolutionary* if the frequencies of different types at a given time is largely explained as a function of their frequencies at earlier time steps (as encapsulated in the Price equation [31,32]). An evolutionary population is subject to *natural selection* if the items exhibit variation, faithful transmission of information through time (heritability), and differences in fitness. Within natural selection, a population is *replicative* if heritability is secured by some form of replication.

The economic theory of long-run growth [4–7] often portrays the development of a single ‘economic system' transforming over time, with faster growing incarnations of the system superseding slower growing ones, thanks to stronger reinforcing feedbacks of endogenous growth. However, the human world contains a population of different types of system whose proportions have changed over time—e.g. industrialized capitalism still coexists with agrarianism and some foraging. Hence, we focus on population thinking, specifically evolutionary populations of systems that exhibit variation but are not replicative. Instead of replication we focus on variation in their feedback loops giving rise to differential spread and persistence, affecting ‘fitness' in the sense of dominance over space and/or time. There are several mechanisms of ‘heritability’ by which feedback information could be faithfully transmitted through time, but this is a point of debate and research [3]. Hence, we remain agnostic about whether we are dealing with a form of natural selection, noting that cultural evolution theory already includes other mechanisms which are not forms of natural selection—notably, humans intentionally produce variation in pursuit of specific goals [30]. We do deal with a dynamical form of ‘stability-based sorting'—or ‘survival of the stable' [33]—the general principle that stable systems tend to accumulate and predominate over time [34]. We interpret this here as more rapidly spreading systems tending to predominate (so long as they are stable in the sense of retaining their identity over time). For example, autocatalytic feedback cycles can rapidly come to dominate a network, accumulating more autocatalytic cycles as they do so [35]—although they are vulnerable to parasitism [36].

Over the past 2 Myr, human innovation has played a key role in reinforcing feedbacks that affected which systems came to dominate and transform the world. Innovation is often portrayed as random (passive), just scaling with (human) population size—i.e. a larger population has a greater chance of producing inventors and inventions [37]. However, innovation is also contingent in that it scales with prior knowledge and innovations [38], making existing knowledge and technology relevant populations. Innovation is also often intentional (active), scaling with education, triggered by problems that need to be solved, and sometimes undertaken for prestige and social reward [20,39]. This is a far cry from random mutation as a source of variation in biology. The contingency of knowledge and technology is one source of a ‘ratchet effect' whereby cultural evolution can become hard to reverse beyond a certain point [40]. Irreversible ratchet effects are a way that human systems can accumulate complexity over time, building upon previous systems. ‘Cumulative cultural evolution' describes how human cultures can accumulate modifications over time, resulting in complex traits that no single individual could invent [41], such as agricultural systems [42]. Humans are also great niche constructors, altering their ecological and developmental environments in ways that feed back to affect their genetic and cultural evolution [43], agricultural being a prime example [42]. Such gene-culture coevolution can sometimes generate strong reinforcing feedback resulting in ‘runaway' cultural niche construction [44].

Systems can interact spatially, exchanging genetic and cultural information and sometimes engaging in conflict. Differential spread of systems across space can occur through several mechanisms. Diffusion is the tendency for anything to move from a region of higher concentration to one of lower concentration (and in its general form is passive not purposive). For humans, in the absence of geographical barriers, denser populations will tend to spread into less densely populated regions (demic diffusion). Reinforcing feedbacks may promote spread by increasing population density (triggering diffusion) and/or by involving self-reinforcing spreading processes, such as fires or disease vectors. Technology [45] and other cultural items (e.g. ideas, languages) may also ‘diffuse' within and between systems (cultural diffusion), but in these cases of cultural transmission of information, adoption (or not) is often deliberate. Where underused resources are available somewhere and this opportunity is communicated to others it can reinforce dispersal there. Where natural selection applies it tends to favour dispersal, even in a uniformly populated world, to minimize competition with relatives [46]. There are also deliberate, aggressive mechanisms of differential spread, including waging wars [47]. These may result in one human system subsuming another, assimilating its people, goods and innovations (a form of recombination mechanism).

Armed with these general principles we now turn to identifying the reinforcing feedback cycles involved in past human revolutions.

## 3. Palaeolithic fire use

**Figure 1.**
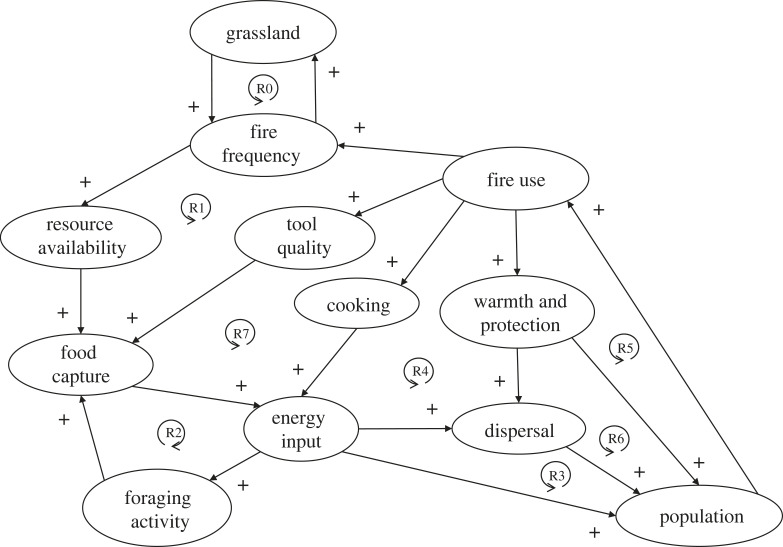
Reinforcing feedback loops in the human use of fire. Solid lines with ‘+' signs denote direct relationships. ‘R' denotes reinforcing (positive) feedback loops, which are described in the text.

The first human revolution was the intentional use of fire, which marked the beginning of a social metabolism (the collectively organized extension of energy and material use beyond biological needs) [48]. Different innovative uses of fire as a technology triggered reinforcing feedbacks across scales (figure 1). Much of early human evolution happened in or near savannah ecosystems in Africa, where existing self-amplifying feedbacks involving grasses promoting fire and herbivory and thus excluding trees were crucial to the expansion and maintenance of the savannah state [3] (figure 1; cycle ‘R0'—where ‘R' is used throughout to denote reinforcing feedback). Sub-Saharan grasslands expanded until approximately 1.8 Ma and their associated fires improved resource availability for foraging animals—including early hominins [49]. It is hypothesized that they learned to transport natural fire to expand burned area and resource availability in this interval [49], thus increasing food capture, energy input, population and fire use (R1). Resulting improvement in diet, including naturally cooked food, has been argued responsible for reductions in tooth size, increased mobility, and thus early dispersal of *Homo erectus* approximately 1.9 Ma [49,50].

Archaeological evidence for intentional, controlled use of fire (in hearths) appears later approximately 1.5–1 Ma [51]. Cooking detoxifies food providing greater food diversity and significantly increases food energy input *per capita*: to a factor of 2–4 above average physiological energy demand [52]. This could support greater hunting and gathering activity and food capture (R2), greater human population and fire use (R3) and dispersal (R4) [46]. Controlled fire also provided warmth and protection from predators reducing morbidity and mortality (R5). At larger scales, use of fire for warmth probably facilitated human dispersal to colder climates [53], increasing global population (R6). Around 400 ka, the archaeological signal of fire becomes geographically widespread consistent with widespread cultural diffusion of this technology [54]. Later (by approx. 165 ka) fire was used to manufacture improved tools [55], thus further increasing food capture and energy input (R7). With fire under control, its use in landscape modification could increase, interacting with ecological reinforcing fire feedbacks (R0) to facilitate (further) transition of forest to grassland and savannah, further increasing resource availability (R1). Deliberate conversion of woodlands to grasslands by anthropogenic fire is seen from approximately 40 ka in Africa [56]. In drier Australia, Aboriginal fire management involving frequent small-scale hunting fires buffered the landscape against large-scale fires started by lightning strikes, increasing vegetation heterogeneity, mammal diversity and resource availability [57,58].

Considering these feedback cycles (figure 1) in an evolutionary light: small-scale reinforcing feedbacks (R2–R5) would have contributed to fire-using groups attaining higher population density than non-fire-using ones and thus tending to diffuse at their expense. There could also be group selection based on differential spread of fire-using groups [9]. Large-scale reinforcing feedback from using fire in landscape modification (R1) amplified pre-existing feedback (R0) which already involved multiple ecological participants, thus creating and enhancing the spread (R4, R6) of the first social-ecological system [59]. Spread of the fire cycles continued after the advent of agriculture. Today all human cultures depend on fire and many still live in fire managed landscapes.

Although technological progress and increasing population can be reciprocally reinforcing in hunter–gatherer societies, it is heavily constrained by ecosystem carrying capacity, consistent with a lack of evidence for endogenous growth in foraging societies (in the Standard Cross Cultural Sample dataset) [21]. Despite fire use, foraging societies still typically needed large areas (and therefore had low population densities) because most natural biomass is not edible to humans. However, in places where natural resources were concentrated, higher population densities and more complex social structures, including settlements, handcraft, trade and social stratification could be supported [60,61]. Settlement in turn may have facilitated the next revolution.

## 4. Neolithic agricultural revolution

The Neolithic revolution was the transition from hunting and gathering to agriculture as the predominant mode of subsistence. Agriculture originated at least 6–10 times independently during the Holocene and spread to many (but not all) cultures. It ultimately increased social metabolism [48]—including the conversion of solar energy into consumable calories, and (via biomass) into heat, mechanical power and chemical transformation (metallurgy)—but this was not the case initially.

Domesticating plants and animals was easy [62], but early farming often had lower calorific return on investment than foraging [63,64], posing a puzzle as to what caused agriculture to become dominant. At low human population density, foraging is favourable, but as population density increases there are diminishing returns of additional foraging labour owing to resource constraints [65]. By contrast, for early farmers with abundant land available for conversion, there would have been relatively constant returns to labour [65]. Hence at a critical population density some agricultural activity could begin alongside foraging [65]. Declining productivity of foraging, increasing productivity of agriculture, or population growth could trigger this reversible transition (trans-critical bifurcation point) [65]. Its reversibility is consistent with historical reversions from farming to foraging [61,66], and with a variable mix of farming and foraging activities among pre-industrial societies [61,62]. Crucially, however, farming can support higher human population density than foraging, because it increases the density of edible species. Hence if population density increased further to the point it exceeded the foraging carrying capacity of the surrounding ecosystem, agriculture could become irreversibly locked in through a ‘ratchet effect' [65].

**Figure 2.**
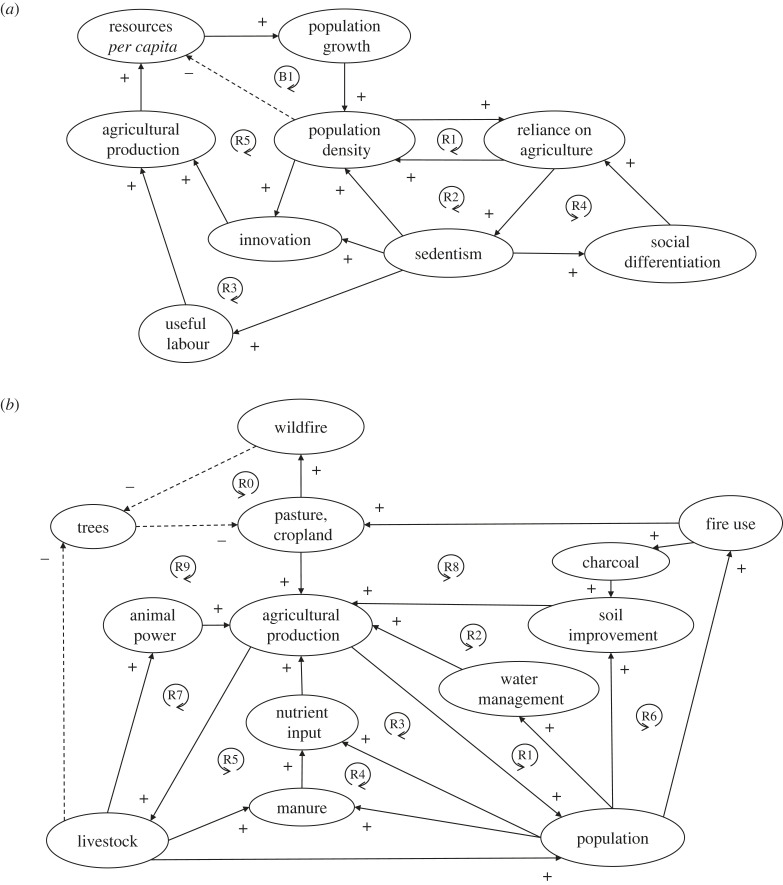
Reinforcing feedback loops proposed to have propelled the Neolithic agricultural revolution. (*a*) Feedbacks behind the establishment of agriculture. (*b*) Feedbacks behind the endogenous growth of agrarian systems. Solid lines with ‘+' signs denote direct relationships, dashed lines with ‘−' signs denote inverse relationships. ‘R' denotes reinforcing (positive) feedback loops, ‘B' balancing (negative) feedback loops. Numbered loops are described in the text.

Once sedentism and farming began, several reinforcing feedbacks could have brought population density past this irreversible tipping point of agricultural lock-in [21,62] (figure 2*a*; ‘R1'): sedentism reduces limitations on family size imposed by a nomadic lifestyle, increasing population density and reliance on agriculture (R2). Children can be put to useful work in farming (but rarely in foraging), increasing productivity (R3) [62]. Wealth can also be more easily accumulated (as possessions do not need to be carried around) potentially producing social stratification, which includes demands for luxury food (R4) [62]—although some societies actively countered this [61]. Crucially, sedentism (affording more time) and population growth (producing more inventors) can trigger innovation and technological improvement [4,62,63], increasing agricultural productivity and population density (R5) [21]. This endogenous growth feedback (R5) even has a quantitative estimate of its gain factor (approx. 0.25) from comparing pre-industrial societies [21]. It would have interacted with the inherent reinforcing feedback in population growth, and the damping (Malthusian) feedback that increasing population reduces resources per capita (B1).

Accepting the evidence that increasing population density spurs technological innovation [4,63], the question becomes; what were the technological innovations and investments in landscape improvement [67] that (transiently) boosted agricultural productivity and population density? Several reinforcing feedbacks of agricultural *intensification* can be identified (figure 2*b*): water management systems e.g. storage, canals, irrigation channels, increased water input [68] (R1). Soil improvement included stone clearance and terracing (to control erosion) [69] (R2). Nutrient addition included the use of natural fertilizers (e.g. guano) [70] (R3). Nutrient recycling began with the return of human excreta to fields as fertilizer (night soil) (R4). Recycling of animal manure and urine added to a highly productive, self-perpetuating system [71] (R5). Addition of charcoal to soils—creating ‘anthropogenic dark earths'—aided the retention of water and nutrients [72] (R6). Draft animal power improved efficiency and productivity [73] (R7).

Reinforcing feedbacks of agricultural *extensification* can also be identified, which built on existing feedbacks (figure 2*b*): productive agricultural area was increased through the use of fires to clear forests e.g. in the pre-Columbian Amazon approximately 4.5 ka [74] and New Zealand approximately 1 ka [75] (R8). Domesticated grazers then helped maintain pasture by eating tree saplings (R9). In many climates, the cleared land increased natural wildfire excluding trees (‘R0', as in figure 1). Pastoralism is a form of extensification to lands unsuitable for crop growing, which can also be reinforced by intentional fires (R8) and the domesticated animals themselves maintaining more productive landscapes (R9) [76]. Concentrations of manure in the pastoralist landscape (e.g. around watering holes) can also produce long-lived islands of productivity and nutrition [77] (R5).

Once dense farming populations were established, they would have tended to diffuse at the expense of less dense foraging ones [78,79]. Reinforcing feedbacks of extensification (figure 2*b*; R8, R9, R0) could also propel the spread of farming across space, creating persistent changes in landscape and reducing land and natural resource availability for foragers (although it might invite them to plunder farms). Spread of farming traits by cultural diffusion would have depended on the relative productivity of foraging (which could be superior). Where both ways of living were equally productive, the persistence of agricultural landscape modifications and the irreversible lock-in to farming would have tended to cause farming to spread at the expense of foraging. Data from Europe suggests slow genetic (demic) diffusion dominated over faster cultural diffusion [78,79].

Considering these feedback cycles in an evolutionary light: some are within social systems (figure 2*a*; R2, R4), but most involve other species (domesticated and wild) and environmental variables (figure 2*b*). Their success (or otherwise) in amplifying the population of farmers was a property of the feedback loops, dependent on other species and abiotic variables. For example, persistence and spread of pasture created in an originally forested region could depend on both domesticated herbivores (R9) and altered fire frequency (R0).

Productive agriculture was a necessary condition for the next revolution [18].

## 5. Rise of complex states

The first complex states started to emerge abruptly from approximately 7 ka onwards, following periods of conflict and limited population growth (or even decline) [80] (possibly linked to climate or environmental degradation). Once established, they were propelled by reinforcing feedbacks of innovation, including increasing governance complexity boosting agricultural productivity [18], and specialization (division of labour) improving performance [3]. Thanks to these feedbacks, agricultural states are estimated to have had three times the population density of non-state-occupied agricultural regions [81]—enabling their diffusion. In addition, agricultural productivity improvements supported the maintenance of armies and associated warfare [18], improving the competitive advantage of farming states over less productive farming groups, and of larger states over smaller ones. This reinforcing feedback produced a ‘competitive ratchet' [82], in which war was a key driver of increasing social complexity [18,83].

**Figure 3.**
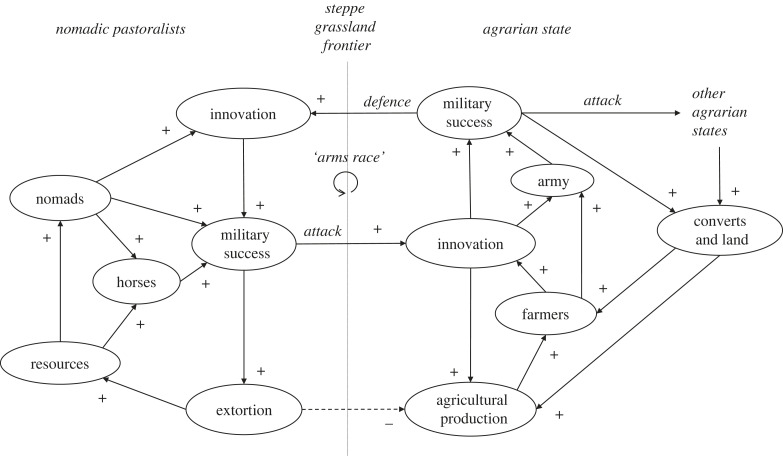
Feedback loops proposed to have propelled increasing state complexity at steppe grassland frontiers. Solid lines with ‘+' signs denote direct relationships, dashed lines with ‘−' signs denote inverse relationships. Here individual loops within polities are not enumerated but the core ‘arms race' reinforcing feedback between federated nomadic pastoralists and agrarian state is denoted. (Also note the aggressive interaction between the focal agrarian state and other agrarian states, which happens across different geographical boundaries, and is also subject to reinforcing feedback.)

Geography was also important. China's tendency towards early and persistent political unification, in contrast with Europe's protracted polycentrism, can be explained by the existence of a core region of highly productive agricultural land in northern China, whereas Europe's productive lands are divided by topographical barriers [84]. Farming states and confederations of nomadic pastoralists repeatedly came into conflict at steppe grassland frontiers, where domesticable horses provided a key military technology for the nomads enabling them to conquer and extort resources from the farmers [85]. This generated reinforcing feedback between military technological innovation on both sides, increasing agrarian state complexity, size and resources that could be plundered [81,85] (figure 3). This reinforcing feedback can explain the expansion to extraordinary size of some old world states [81]. However, those very large states typically did not have correspondingly long lifetimes, because within them escalating elite conflict for control of dwindling resources led to instability and civil war [81]. The inter-polity reinforcing feedback (figure 3) repeated when European settlers introduced horses to Native American communities, who then rapidly assimilated them into trade networks, hunting practices and resistance against the invaders (e.g. the Comanche) [3].

Thinking evolutionarily: crucial reinforcing feedbacks had now shifted further to the social realm, but still depended on other ecological participants in ever more productive agricultural systems. A tension between spread and persistence emerges: reinforcing feedbacks accelerating the spread of a new system may end the persistence of incumbent systems but are not in themselves a source of stability for the spreading system. Sometimes they may trigger internal reinforcing feedbacks that shorten persistence of the spreading system—e.g. growing social inequality and internal conflict [81]. However, while specific states and levels of social complexity may be transient, underlying feedback structures (e.g. figure 3) appear persistent.

With agriculture predominant, population and technology remained stabilized in a ‘Malthusian regime' [21], where increasing resources *per capita* caused population growth but that diluted resources *per capita* (damping feedback), leading to minimal long-term growth in population or well-being [5]. Several conditions are hypothesized as necessary for the next revolution: the emergence of agrarianism then mercantilism provided the foundations for modern capitalism—an economic system based on the private ownership of the means of production and their operation for profit. The ‘Age of Discovery’ decimated indigenous populations of the Americas and established persistent geographical differences in economic prosperity, with richer western European nations extracting labour (slaves) and raw materials (e.g. cotton, sugar, tobacco) from poorer nations in a reinforcing cycle [86]. Britain had high wages and cheap access to energy (coal), which gave strong incentives to invent technologies that substituted capital and coal for relatively costly labour, in order to compete globally [87]. In 1715–1750, England also saw stable weather, good harvests and low food prices, which increased demand for industrial goods.

## 6. Industrial Revolution

The Industrial Revolution has been defined as the sustained increase in the rate of growth of total and *per capita* output at a rate which was revolutionary compared with what went before [88]. Also known as the ‘Great Divergence', the abrupt and sustained increase in growth rates of gross domestic profit (GDP)/capita occurred around 1800 in Great Britain [89], while the transition to new manufacturing processes spanned roughly 1760–1840. The Industrial Revolution radically increased social metabolism through accessing and using fossil fuel energy [48]. During it, Malthus issued his famous (1798) warning [90] that arithmetic growth of food supply would limit exponential growth of population—and thus labour supply for economic growth. However, actually in the Industrial Revolution, technological progress drove accelerating growth of output faster than population growth diluted resources *per capita*—representing a switch to a ‘post-Malthusian' growth regime [5]. Here, we focus on the reinforcing feedback cycles that propelled the extraordinary growth of output [20], rather than the ultimate causes of why the Industrial Revolution started where and when it did, recognizing that there are a diversity of hypothesis for both, which remain contested [87].

**Figure 4.**
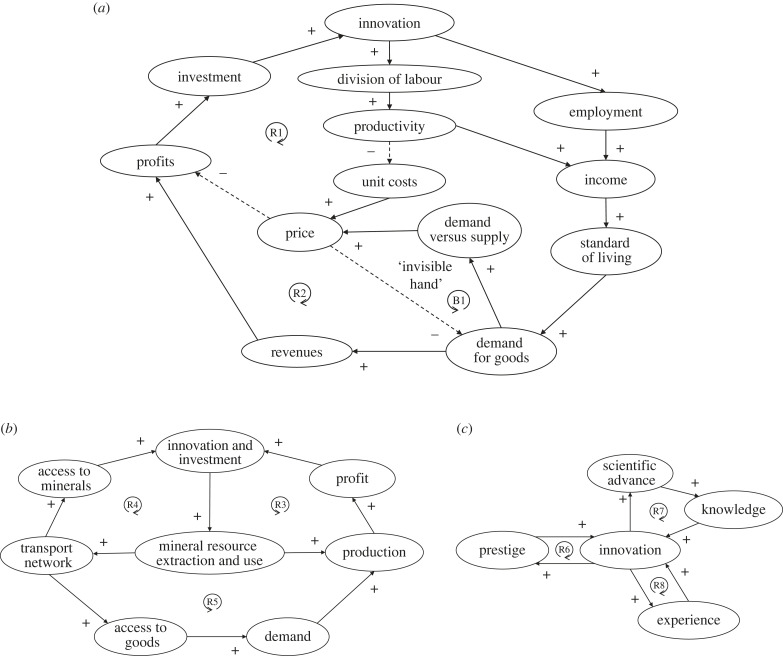
Feedback loops proposed to have propelled the Industrial Revolution (inspired in [20]). (*a*) Adam Smith's theory of economic growth. (*b*) Energy and mineral resource feedbacks. (*c*) Innovation feedbacks. Solid lines with ‘+' signs denote direct relationships, dashed lines with ‘−' signs denote inverse relationships. ‘R' denotes reinforcing (positive) feedback loops, ‘B' balancing (negative) feedback loops. Numbered loops are described in the text.

Adam Smith's theory [91] of economic growth is central to most descriptions of the Industrial Revolution and can be portrayed in terms of two key reinforcing feedbacks [20] (figure 4*a*). On the supply side (R1), investment (by capitalists of business profits) in innovation and new and more specialized methods of production involving increased division of labour, increases productivity and output, reducing unit costs and price and increasing profits, supporting further investment. On the demand side (R2), increasing employment, income and standard of living increases demand for goods, revenue and profit, supporting further investment. Balancing feedback (B1) operates if demand exceeds supply causing delays and pushing prices up, thus reducing demand (or if supply exceeds demand lowering prices thus increasing demand). However, if supply keeps up with demand, demand stays high, and operating costs remain low, growth can continue—at least until increased competition for labour, employment and wages, and declining profits lead to a stationary state [91]. The demand side reinforcing feedback (R2) is questioned by evidence that average real wages and standard of living for workers remained low during the Industrial Revolution [92,93]—but this can be understood as the result of decreasing wages for those working in the existing handicraft economy and increasing wages for those working in the new industries [87].

When Adam Smith wrote in 1776 [91], he was concerned that competition for land would constrain industrialization, assuming wood as an energy source—but then cheap coal took over. The supply and use of fossil fuel energy and mineral resources—notably coal and iron—were key to reinforcing feedbacks propelling the Industrial Revolution [20] (figure 4*b*). Investment in innovation in energy and material extraction led (through discoveries, economies of scale, and technical progress in energy conversion) to cheaper useful energy (exergy) supply, which substituted for labour and capital, lowering production costs (increasing productivity), increasing profits and demand for goods, and stimulating further investment, innovation and substitution of mechanical power for human (and animal) labour [94] (R3). The sheer mass of minerals involved required investment in a transport network (e.g. to connect mines and factories), whereas organic materials beforehand did not. This increased access to mineral resources, fuelling further investment, innovation, extraction and use [95] (R4). The transport network in turn got used to expand the market both for industrial goods and for organic goods (e.g. cotton), thus increasing demand, production, profits, investment and innovation [95] (R5). Overall economic efficiency increased to a point where investment in industry could yield as good (or better) return as investment in the land.

Deliberate innovation and entrepreneurship, supported by a ‘gentlemanly' culture of trust and cooperation [96], and including available credit to invest with no immediate return, were key to the Industrial Revolution. They relied on an underpinning Enlightenment foundation of existing knowledge and inventions [97]. Schumpeter [98] famously described innovation—‘a feat not of intellect, but of will'—as a uniquely disruptive process that creates an inherent instability in capitalism. Innovation increased productivity, creating whole new products and markets, and happened fast enough to keep profits rising. Innovation triggered reinforcing feedbacks (figure 4*c*) of increased prestige for the entrepreneurs encouraging further innovation [39,96] (R6), advancement of science and knowledge providing a greater foundation for further innovation [97] (R7), and learning-by-doing (R8).

Considering these feedback cycles (figure 4) in an evolutionary light: in ‘Smithian growth', the reinforcing feedback of specialization (division of labour) improving performance (R1) is a generic one with a precedent in ecology [3]. Its operation at the level of businesses (as groups) could be part of spread/persistence-based selection between businesses. The reinforcing feedback of a growing and consuming middle class (R2) was economy and society wide, containing diverse (human) components, and thus its spread is hard to frame in terms of individual or group selection. The reinforcing feedback of increasing energy supply fuelling production and further resource extraction (R3) has parallels in biosphere history [48,99], and was also economy-wide. Reinforcing feedbacks of transport network construction (R4, R5) were somewhat unintended and society wide. Entrepreneurial innovation was clearly intentional and the feedbacks reinforcing it (R6–8) are a mix of psychological and social, ranging across scales. Overall, key feedbacks became divorced from ecology but clearly depended on energy and material resources.

**Figure 5.**
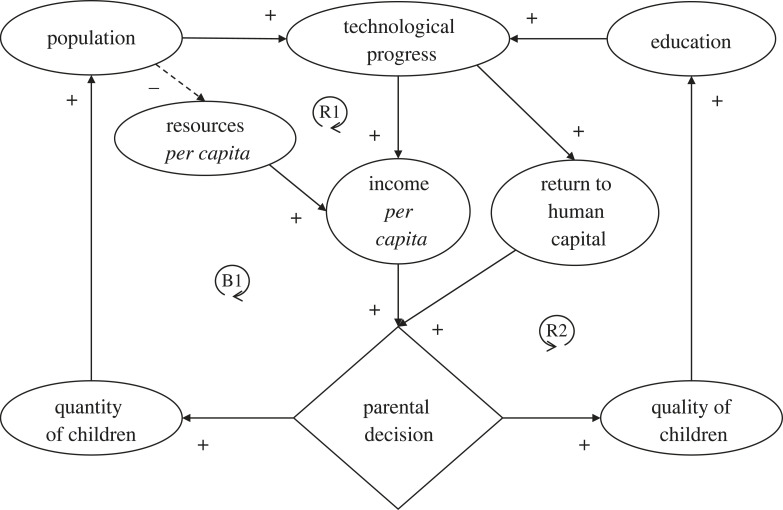
The switch in feedbacks to a modern growth regime. Solid lines with ‘+' signs denote direct relationships, dashed line with ‘−' sign denotes inverse relationship. ‘R' denotes reinforcing (positive) feedback loops, ‘B' balancing (negative) feedback loops. Numbered loops are described in the text.

The Industrial Revolution was followed by a demographic transition to a modern growth regime characterized by a negative relationship between income and population growth rate [5] (figure 5). This can be understood in terms of a switch in parental decisions [5]: technological progress provided greater income allowing households to spend more on raising children—which during the Industrial Revolution still resulted in them having more children (R1)—but it also raised the rate of return to human capital inducing parents to reallocate increased resources from quantity to quality of children. This switch is seen in a dramatic rise in schooling in Europe over the nineteenth century [5]. Better educated children in turn tend to accelerate technological progress, producing reinforcing feedback (R2), which is linked to declining population growth. Declining population growth in turn reduced the influence of the Malthusian damping feedback (B1) of diluting finite resources. Increasing education rather than increasing population became a driver of ongoing technological progress and growth. Although it took the UK nearly a century to halve fertility rates from more than six to less than three children per woman, subsequent countries have done so progressively faster—many in less than 25 years, and China in only 11 years (before the introduction of the one-child policy) [100,101].

Thinking evolutionarily, voluntarily reducing the number of ones' descendants is completely at odds with natural selection (even if they have a longer and more prosperous life). ‘Cultural evolution' has clearly become (it if wasn't already) fundamentally different to biological evolution. Purposeful innovation supported by investment in education, further shows that actual learning has superseded the learning algorithm of natural selection [102]. The differential spread of wealth has become more important than that of people—societies in the modern growth regime dominate the world in terms of wealth, power and ideology, if not (yet) population or territory. It is maintained and reinforced by the richest nations appropriating resources (materials, energy, land and labour) from less prosperous nations, exacerbating between-country inequality [24]. While an alternative planned economic system spread temporarily, its largest example in terms of area and power—the Soviet Union—failed to persist, catastrophically. (China while nominally a ‘socialist market economy' contains key elements of capitalism.) The modern growth regime is reinforced by the increasingly efficient conversion of fossil fuels to useful energy (exergy), which lowers prices and increases production [94]. More recently it has also been reinforced by information and communications technology substituting knowledge for labour, capital and energy [103]. Thus, key feedbacks still depend on energy and materials, but some decoupling has begun.

## 7. Taking stock

Globally the ongoing spread of the industrialized modern growth regime is what continues to accelerate us into the Anthropocene. However, it is threatening itself in at least two ways: the growing consumption of non-essential goods, services and associated resources is causing climate change, loss of nature and disruption of humanity's life-support system [104] which is starting to trigger human conflict [105]. At the same time, appropriation of resources by the richest is exacerbating inequality between nations [24] and within some of the richest nations [106], producing geopolitical tension, social unrest and conflict [107] (despite average wealth increasing). Clearly, spread is not equivalent to persistence—as with historical empires, the fastest and furthest spreading system may also be the one prone to most rapid internal breakdown. However, waiting for the industrialized growth regime to bring about its own collapse is not an attractive option for those within it. Rather humanity needs to transform towards sustainability without losing some hard-won gains.

Having used the lens of ‘spread of the cycles' to help understand how current unsustainable human systems became dominant, we now use it to identify some qualities that more sustainable systems need to possess to rapidly displace them. Given the ongoing spread of fossil-fuelled industrialization and associated inequality, there is an immediate need to *out-spread* its cycles with some based on (more) sustainable and socially just behaviours and technologies. However, this sets up a clear tension: promoting short-term ‘green growth’ (spread) in pursuit of longer-term sustainability (persistence) is an oxymoron if indefinite economic (GDP) growth is impossible on a finite planet [108–110]. Evidence suggests GDP growth cannot be decoupled from useful energy (exergy) use, and only relative decoupling from material consumption has been observed [110,111]—although absolute decoupling of greenhouse gas emissions has begun to occur in some industrialized nations (thanks to transitioning to renewable energy and decreases in energy use) [112]. Furthermore, if promoting ‘green growth' perpetuates existing patterns of ecologically unequal exchange [24], it will not offer a path to social justice [113], let alone inter-species justice [114]. ‘Green transformation' policies that realign growth to sustainable development principles are beginning to be applied, particular in the energy sector, in pursuit of more socially just outcomes [113]. However, a more fundamental ‘green revolution' of structural transformation may be needed to achieve sustainability [113,115].

Given these considerations, we next consider the specific example of accelerating to net zero greenhouse gas emissions by mid-century before turning to the broader challenge of justly transforming to sustainability.

## 8. Seeking the next cycle

If more sustainable systems are to out-spread fossil-fuelled industrialized systems, they are going to need strong reinforcing feedbacks behind them. Such ‘virtuous cycles' [116] of uptake of sustainable options, if they get strong enough, can become self-propelling [117–121]. In the Global North, already in the modern growth regime, the question is: how can more sustainable systems spread at the expense of incumbent systems (i.e. displace them)? Whereas in parts of the Global South yet to industrialize or enter a modern growth regime, the question is: how can more sustainable systems spread more effectively than fossil fuelled ones?

**Figure 6.**
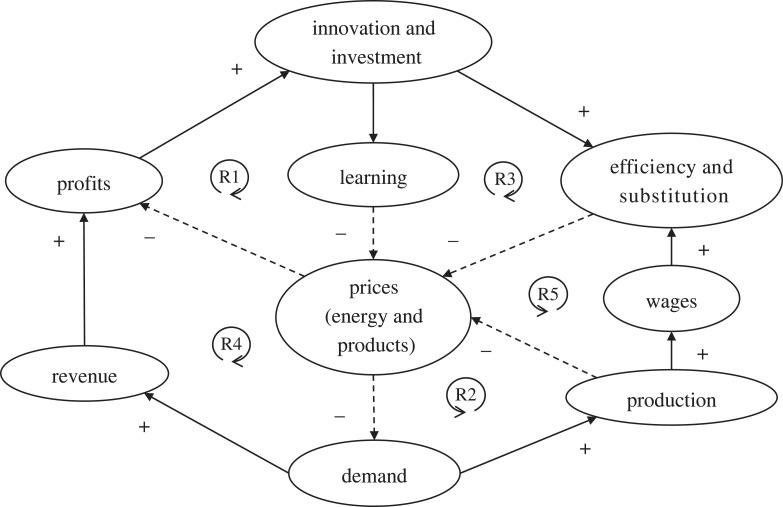
Reinforcing feedbacks in the transition to renewable energy. ‘Efficiency' refers to the (increasing) thermodynamic efficiency of converting energy to useful work (exergy) leading to (increasing) ‘substitution' of exergy for labour. Solid lines with ‘+' signs denote direct relationships, dashed lines with ‘−' signs denote inverse relationships. ‘R' denotes reinforcing (positive) feedback loops, ‘B' balancing (negative) feedback loops. Numbered loops are described in the text.

Taking decarbonization as an example, the energy sector comprises approximately 75% of global emissions with power (electricity) generation approximately 25%. Renewable energy has just started to out-spread and displace fossil-fuels in the power sector, with global growth in new renewable energy installation exceeding the growth in electricity demand in 2022 [122], and generating electricity cheaper than new fossil fuel power stations in most of the world [123]. Strong reinforcing feedback loops (figure 6) of learning-by-doing (R1) and economies of scale (R2)—Wright's Law [124]—have made renewable energy markedly cheaper the more that is deployed. Over the last decade, the price of solar power has fallen nearly 90% and of onshore wind power around 70% [125] (whereas fossil fuels have been a similar price for over a century [126]). As the price of renewable power continues to drop this will incentivise electrification wherever possible, and where not, its use to produce fuels (e.g. hydrogen, ammonia). Thanks to reinforcing feedbacks reducing battery costs by nearly 90% over the last decade [125], electric vehicles are already starting to displace internal combustion engine vehicles in several major economies [118]. Reinforcing feedbacks across sectors are also emerging, for example, cheap batteries provide electricity storage reinforcing the transition to renewable energy, which in turn reinforces the transition to electrifying mobility [118]. The spread of renewable power and electrification increases the thermodynamic efficiency of energy conversion to useful work (exergy), lowering prices of energy, goods and services (R3), increasing demand, revenue, profits and stimulating further investment (R4), and also increasing production, wages and substitution of exergy for labour (R5) [94]. It is projected to generate net employment, reinforcing growth [122]. These strong reinforcing feedbacks mean the more that gets invested in the energy transition, the faster it will unfold, saving us all money [126].

More broadly, reinforcing feedbacks of sustainable technology innovation (recall figure 4*c*) can be triggered by environmental regulation increasing productivity, profit and efficiency [127]. Already approximately 30% of global innovation and trade in ‘green' technologies is coming from the Global South [128,129], where the declining cost of renewables suggests ‘green' technologies have the potential to spread faster than fossil-fuelled ones. Constraints on raising finance for green projects in the Global South are holding things back [130], but the energy transition will improve the balance of trade for most least developed countries (as they will e.g. no longer have to import fuel as well as vehicles), producing reinforcing feedback of more capital to invest. If the energy transition thus helps lift countries out of poverty, abrupt shifts to lower fertility rates should follow, contributing towards long-term sustainability.

The bigger challenge is: how to transition from maximizing growth (of production) to maximizing persistence (sustainability)? Key to this is for human systems to deliberately shift emphasis from reinforcing (positive) to damping (negative) feedbacks. At a minimum humanity will need to get better at correcting its mistakes [131].

If the world manages to massively reduce greenhouse gas emissions through ‘green growth' or ‘green transformation', overall material use will still probably increase—as absolute decoupling from GDP has never been observed [111]. Switching to a material recycling ‘circular economy' is a prerequisite for sustainability [131] and possible in principle: renewable energy from the Sun can power a near perfect material recycling system without violating the second law of thermodynamics—just as it does in the biosphere—so long as there is a reservoir of inactive wastes alongside the materials in active use [23,99]. This is inhibited in practice by the equating of social ‘progress' with production, i.e. growth that maximizes consumption (GDP), without any consideration of natural capital or social justice. It remains thermodynamically possible that knowledge (information) could continue to grow powered by a (growing) sustainable supply of useful energy (exergy) in a steady-state material recycling economy [23,94,103]. However, instead the modernist notion of ‘progress' equated with production may need to be abandoned in favour of a different notion of prosperity anchored in a new worldview that redefines what we depend upon—and need to politically defend—as the Earth's life-support system [115]. The resulting shift in cultural values towards cherishing our life-support system, quality of (all) life, and ‘Earth system justice' (including multiple forms of social justice) [114], would be profound indeed.

This may seem unrealistic, particularly given the thus far modest emergence of a new ‘ecological class', and its struggles to mobilize popular support [115]. However, there are powerful precedents of attitude shifts that are hard to explain from the strive for economic progress, wealth or power. For instance, despite formidable economic interests, the trans-Atlantic slave trade that flourished for centuries became globally abandoned within a few decades [132]. After persisting for about a millennium, the practise of foot-binding in China was eventually abandoned within one generation [133,134]. Smoking in public places was recently rapidly abandoned in most developed countries [135]. Traditionally high fertility rates have halved within a generation in many countries [100,101]. Drivers of such change of dominant attitudes often include growing awareness of dissonance between the practice and broader values held by society, and they typically involve cascading co-evolving change in attitudes and regulations. The transitions are typically preceded by long periods during which social movements and policy efforts pushed for change (seemingly) unsuccessfully.

At heart this, and coming generations, face a deep cultural evolutionary challenge: given a history of innovation generating reinforcing feedbacks that out-spread existing systems, humanity needs to evolve to innovate to maximize persistence (sustainability). Happily, there is nothing un-evolutionary about such a revised goal—as the phrase ‘survival of the fittest' encapsulates.

## 9. Outlook

Our aim has been to help explain what *propelled* the human ‘revolutions' that started the Anthropocene and to offer some clues as to what could speed humanity out of trouble. This is not the same as explaining what *triggered* (or could trigger) revolutionary change, which may be owing to internal evolution, external forcing or a particular perturbation of a complex adaptive system. However, for change to become large-scale, abrupt and/or irreversible—and hence ‘revolutionary'—in any complex system, usually requires the presence of reinforcing feedback loops that can become strong enough to be self-propelling. Hence, we have sought to synthesize existing hypotheses as reinforcing feedback loops, or parts thereof. This complements existing approaches by bringing together mechanistic proposals in a common framework of causal feedback loop diagrams. We also offered a novel framing of the immediate sustainability challenge as one of outspreading the incumbent cycles of the industrialized growth regime.

There are several ways to develop and apply these ideas further. Mapping feedbacks is a first step towards developing a process-based, dynamical system model. While data invariably get sparser going further back in time, this presents an opportunity for deeper understanding of the dynamics of more recent revolutions. The Industrial Revolution would seem an obvious target given there are sufficient data for econometric modelling [136]. The rise of complex states [18] and even the agricultural revolution [21] also have sufficient data to identify and begin to quantify key reinforcing feedback loops. For a prospective future ‘green revolution', dynamical system models of innovation feedbacks coupled to the macro-economy already exist [137] with considerable potential to extend their representation of feedbacks and the coverage of sectors.

To strengthen the link to cultural evolution theory requires deeper consideration of whether and how the feedback cycles we identify can evolve. Trying to develop conceptual models of spread/persistence-based selection of cycles should be instructive. At a theoretical level fundamental questions need addressing [3], notably: how is information pertaining to the irreducible properties of whole feedback loops—e.g. their sign and strength—transmitted through time? This whole-system information is not (by definition) contained in the components of the loop but could conceivably be culturally transmitted. This paper is a humble step towards that goal, but what are its predecessors?

To conclude, we have traced the reinforcing feedback cycles whose differential spread propelled the human ‘revolutions' that brought us into the Anthropocene. We have also begun to suggest some new cycles that could help speed us out of trouble. The cycles behind early human revolutions were often comprised of diverse, interconnected, unrelated, human and non-human elements. Over time, the spread of cycles based purely on human elements (albeit diverse ones) became progressively more important. Looking ahead we suggest that non-human elements need to be brought back into the feedback cycles underlying cultures, human notions of progress, and theories of cultural evolution—if humanity is to make another revolutionary cultural shift from maximizing growth to maximizing persistence.

## Data Availability

This article has no additional data.
